# Retinoic Acid Upregulates METTL14 Expression and the m^6^A Modification Level to Inhibit the Proliferation of Embryonic Palate Mesenchymal Cells in Cleft Palate Mice

**DOI:** 10.3390/ijms25084538

**Published:** 2024-04-20

**Authors:** Yue Zhu, Yadong Zhang, Yaoqi Jiang, Hongshi Cai, Jianfeng Liang, Hongyu Li, Cheng Wang, Jinsong Hou

**Affiliations:** 1 Hospital of Stomatology, Guanghua School of Stomatology, Sun Yat-sen University, Guangzhou 510055, China; zhuyue8@mail2.sysu.edu.cn (Y.Z.); zhangyad@mail2.sysu.edu.cn (Y.Z.); jiangyq8@mail2.sysu.edu.cn (Y.J.); caihsh3@mail2.sysu.edu.cn (H.C.); liangjf3@mail3.sysu.edu.cn (J.L.); lihongy5@mail2.sysu.edu.cn (H.L.); 2 Guangdong Provincial Key Laboratory of Stomatology, Guanghua School of Stomatology, Sun Yat-sen University, Guangzhou 510055, China

**Keywords:** cleft palate, m^6^A modification, cell proliferation

## Abstract

Cleft palate only (CPO) is one of the most common craniofacial birth defects. Environmental factors can induce cleft palate by affecting epigenetic modifications such as DNA methylation, histone acetylation, and non-coding RNA. However, there are few reports focusing on the RNA modifications. In this study, all-trans retinoic acid (atRA) was used to simulate environmental factors to induce a C57BL/6J fetal mouse cleft palate model. Techniques such as dot blotting and immunofluorescence were used to find the changes in m^6^A modification when cleft palate occurs. RNA-seq and KEGG analysis were used to screen for significantly differentially expressed pathways downstream. Primary mouse embryonic palate mesenchymal (MEPM) cells were successfully isolated and used for in vitro experimental verification. We found that an increased m^6^A methylation level was correlated with suppressed cell proliferation in the palatine process mesenchyme of cleft palate mice. This change is due to the abnormally high expression of m^6^A methyltransferase METTL14. When using siRNAs and the m^6^A methyltransferase complex inhibitor SAH to interfere with the expression or function of METTL14, the teratogenic effect of atRA on primary cells was partially alleviated. In conclusion, METTL14 regulates palatal mesenchymal cell proliferation and cycle-related protein expression relies on m^6^A methylation modification, affecting the occurrence of cleft palate.

## 1. Introduction

Cleft lip with or without palate (CL/P) and cleft palate only (CPO) are the most common craniofacial birth defects and can occur alone or in association with more than 500 known malformation syndromes. With the advancement of prenatal examination technology, the number of children with severe CL/P has gradually decreased in recent years. Today, more attention is paid to children with CPO, which is not easy to detect during pregnancy. If parents do not recognize the disease in time after birth, it will have a greater impact on their child’s appearance, speech, swallowing, and other functions, and even their physical and mental health. Therefore, determining how to detect and prevent such craniofacial deformities earlier is one of the tasks of clinicians and researchers. Epidemiological and embryological development studies have shown that the pathogeneses of CPO and CL/P are different. CPO is caused by the failure of the two palate processes to fuse at the midline. Approximately 50% of CPO cases manifest in non-syndromic forms [1]. The causes are complex and are affected by both genetic factors and environmental factors, of which environmental factors account for 50% to 80% [2].

Palate development is a complex and continuous process achieved by cell signal transmission, transduction, and regulation under the control of genes. Many transcription factors and their receptors, extracellular matrix, etc., are involved in this process [3]. Any abnormal interference may lead to cleft palate. Numerous studies have confirmed the key role of Mendelian inheritance laws, gene mutations, and single-nucleotide polymorphisms (SNPs) in the pathogenesis of cleft palate. However, the etiological mechanism of non-syndromic cleft palate only (nsCPO), in which the causative gene cannot be diagnosed and is affected by a combination of multiple genes and environmental factors, has not yet been clarified. We believe that normal development of the palate not only relies on the correct spatial and temporal expression of genes, but also relies on the regulation of stable and harmless epigenetic modifications. Epigenetic modification is an important regulatory mechanism for the spatiotemporal expression of embryonic development genes and plays a key role in craniofacial development [4,5,6,7]. Recent studies have found that exogenous teratogenic effects such as smoking, drinking, drug misuse or abuse, nutritional deficiencies, and contact with infections during pregnancy can induce the occurrence of cleft palate by affecting cell DNA methylation, histone modifications, and non-coding RNA [5,8,9,10,11]. In recent years, more and more researchers have focused on the regulatory mechanism of RNA modification on craniofacial development. RNA epigenetic modifications include m^6^A, m^5^C, hm^5^C, m^1^A, pseudouracil, and m^7^G modification, etc. Among them, N6-methyladenosine (m^6^A) is the most abundant chemical modification in eukaryotes, and is involved in the occurrence and development of various physiological and pathological processes such as embryonic development, metabolic diseases, and tumors [12,13,14,15]. It has been found that knocking down METTL3 or METTL14 significantly reduces the self-renewal ability of embryonic stem cells [16]. Ma et al. [17] elucidated the key role of the METTL3/YTHDF1/PSEN1/β-catenin signaling axis in craniofacial development through a zebrafish model. Some scholars have found that m^6^A methyltransferases (METTL3/METTL14) regulate osteogenic proliferation, and differentiation of bone marrow mesenchymal stem cells (BMSCs) may affect bone development and regeneration [18,19,20,21]. However, there are currently few reports on m^6^A methylation regulating secondary palate development.

Based on the preliminary foundation of our research group and the progress of international research, we speculate that environmental factors can regulate the growth and development of the embryonic palate by affecting m^6^A methylation modification, and the imbalance of m^6^A methylation modification levels may lead to nsCPO. This study used all-trans retinoid acid (atRA) to simulate the teratogenic effects of environmental factors, and established a C57BL/6J fetal mouse cleft palate model to analyze the role of m^6^A modification. Primary mouse embryonic palate mesenchymal cells (MEPMs) were obtained for in vitro experiments to analyze the regulatory mechanism of the enzyme methyltransferase like 14 (METTL14) on cell proliferation and the cell cycle. This article aims to provide a new scientific basis for etiology research and the clinical prevention and treatment of cleft palate from the perspective of m^6^A modification.

## 2. Results

### 2.1. Morphological Observation of Two Fetal Mouse Models with Cleft Palate

**Figure 1:**
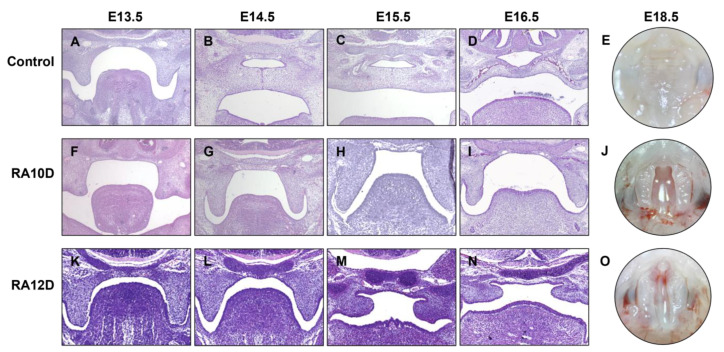
Morphological observation of normal and cleft palate mouse models. (**A**–**D**) HE staining of the normal palate development of embryonic mice in the control group from E13.5 to E16.5 days. (**F**–**I**) HE staining of the palate process development in the RA10D cleft palate group from E13.5 to E16.5 days. (**K**–**N**) HE staining of the palate process development in the RA12D cleft palate group from E13.5 to E16.5 days. (**E**,**J**,**O**) Observation of the palate in the control group, RA10D group, and RA12D group at E18.5 days for embryonic mice under a stereomicroscope.

In this study, we constructed two kinds of C57BL/6J fetal mouse cleft palate models induced by intragastric administration of 0.1 mg/g atRA corn oil suspension on day E10.0 (RA10D) or day E12.0 (RA12D) of pregnancy separately to explore the different morphologies of cleft palate and normal mice, and to find the potential biomarkers that may affect the occurrence of cleft palate. The results are shown in Table 1. None of the embryos in the control group were dead fetuses, and no cleft palate or other craniofacial abnormal phenotypes were found (Figure 1A–E). On days E13.5–E18.5, the palatal shelves in the control group grew vertically on both sides of the tongue. Then, the palatal shelves rose horizontally and grew relatively, and the tongue descended and became flat. The bilateral medial edge epithelial (MEE) contact formed the medial epithelial seam (MES) and gradually subsided, the mesenchyme began to fuse, the bilateral palatal shelves fused to form a complete palate, and osteogenic differentiation began. Finally, a complete palate was formed, with obvious palatal rugae and continuous lines. The experimental RA10D group had an embryonic survival rate of 81.6% (111/136), and the incidence of cleft palate in the surviving fetal mice was 97.3% (108/111).

In this group (Figure 1F–J), the bilateral abnormally small palatal shelves were perpendicular to both sides of the tongue and could not be raised, forming a wide cleft palate model. In another experimental group (RA12D), the survival rate was 93.1% (108/116), and the incidence of cleft palate was 85.2% (92/108). In this group (Figure 1K–O), the shape of the palatal shelves was not obviously abnormal, the shelves could contact each other but could not be completely fused, and a narrow cleft then formed in the middle of the secondary palate.

### 2.2. atRA Upregulated the m^6^A Level and the Expression of METTL14 in the Embryonic Palatal Mesenchyme

**Figure 2:**
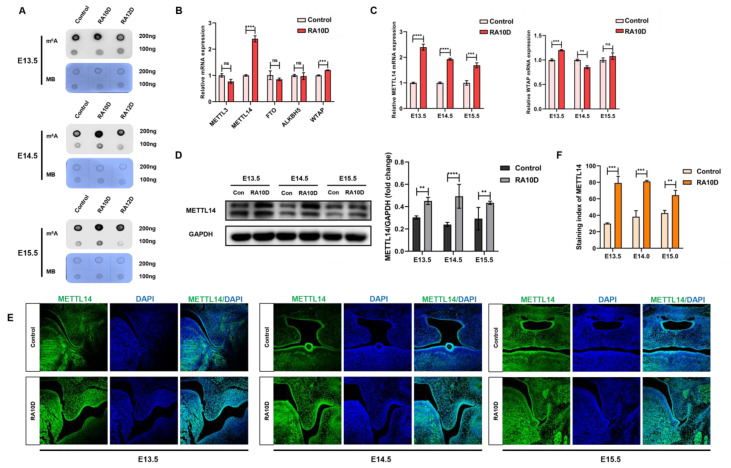
Increased m^6^A level and expression of METTL14 in embryonic palatal mesenchyme were associated with cleft palate. (**A**) Dot blot was used to detect the m^6^A modification level of RNA in the palatal mesenchyme of embryonic mice in the control group, RA10D group, and RA12D group. (**B**) qRT-PCR detection of the expression of m^6^A methylation modification enzymes in the palatal mesenchyme of embryonic mice in the control group and RA10D group. (**C**) qRT-PCR detection of METTL14 and WTAP mRNA expression and changes in the palatal mesenchyme of the control group and RA10D group on days E13.5–E15.5. (**D**) Western blot detection of METTL14 expression in the palatal mesenchyme of embryonic mice in the control group and RA10D group. (**E**,**F**) Immunofluorescence detection and semi-quantitative analysis of METTL14 expression in the palatal mesenchyme of embryonic mice in the control group and RA10D group from E13.5 to E15.5 days (green: METTL14, blue: DAPI) (10× magnification). n.s is considered not statistically significant, ** means *p* < 0.01, *** means *p* < 0.001, **** means *p* < 0.0001.

In order to evaluate the changes in the m^6^A methylation modification level of embryonic palatal mesenchyme tissues with different palate morphological characteristics during the key period of palate development (E13.5–E15.5), we used dot blotting experiments to detect the binding abilities of RNA and m^6^A antibodies. We found that in E13.5/E14.5/E15.5 of the RA10D group, the m^6^A levels of the palatal mesenchyme were significantly higher than those in the control group, while there was no significant difference between the RA12D group and the control group (Figure 2A). We obtained the total mRNA and protein from the palatine process mesenchyme of the control group and RA10D cleft palate group on days E13.5/E14.5/E15.5, and detected and screened out the key enzymes. qRT-PCR found that the m^6^A methyltransferase METTL14 was significantly increased in the palatal mesenchyme of RA10D cleft palate mice (*p* < 0.0001), while the other m^6^A methylase or demethylase enzymes (METTL3, WTAP, FTO, and ALKBH5) had no significant differences (Figure 2B,C). Western blotting found that the protein expression level of m^6^A methyltransferase METTL14 in the palatine process mesenchyme of embryonic mice with cleft palate (RA10D) was significantly higher than that of the normal palate in the control group during the same period (*p* < 0.0001) (Figure 2D). Immunofluorescence staining revealed that the m^6^A methyltransferase METTL14 was abnormally highly expressed in the palatine process mesenchyme of embryonic cleft palate mice (RA10D) throughout the key period of palate development (E13.5–E15.5) (Figure 2E,F). In summary, the level of m^6^A methylation modification in the palatine process mesenchyme of cleft palate mice was abnormally elevated throughout the key period of palate development, and was positively correlated with the expression of METTL14 (E13.5–E15.5).

### 2.3. Environmental Factors Affect the Cell Cycle and p53 Signaling Pathway, Leading to Cleft Palate

**Figure 3:**
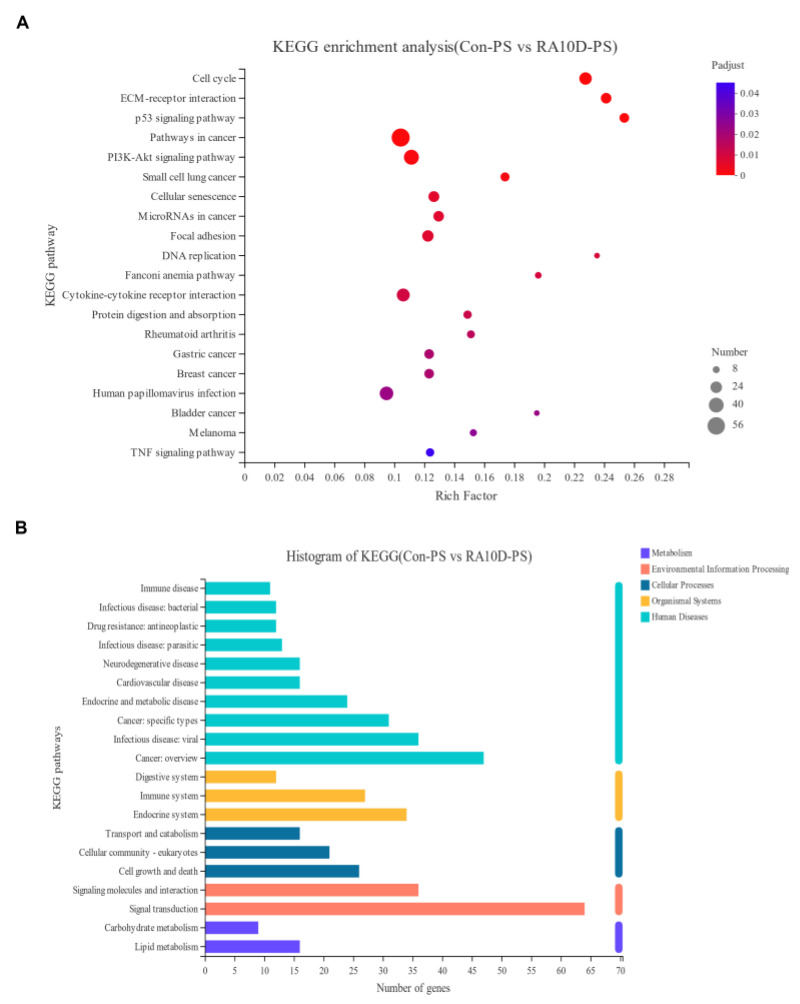
KEGG analysis of differentially expressed genes in the palatal mesenchyme of embryonic mice on day E13.5 between the control group and the RA10D group. (**A**) Functional enrichment analysis scatter plot. (**B**) Functional annotation analysis histogram.

RNA-seq analysis was performed using the palatine process mesenchyme of the control group and RA10D group on day E13.5 to screen differential gene expression, and KEGG functional enrichment analysis was performed to find potential signaling pathways that may affect palate development and relate to cleft palate. RNA-seq showed that atRA mainly regulated palate development by affecting the cell cycle, extracellular matrix interaction, and p53 signaling pathway (Figure 3A,B). Based on our previous research and the RNA-seq results, we focused on genes related to the G1/S phase of the cell cycle and proliferation regulation (Supplementary Figure S1A,B).

### 2.4. Inhibition of Mesenchymal Cell Proliferation in Cleft Palate Mice May Be Related to the Abnormal Expression of METTL14

**Figure 4:**
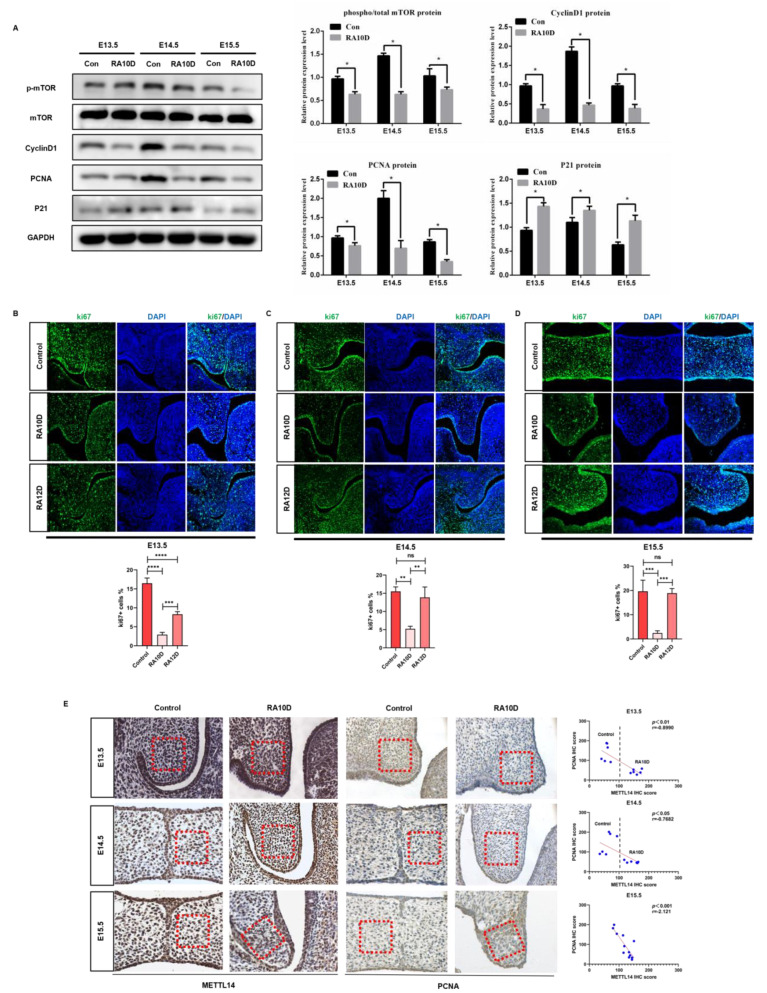
Inhibition of mesenchymal cell proliferation in cleft palate mice may be related to the abnormal expression of METTL14. (**A**) Western blot detection of the proliferation and cell cycle-related differential genes of palatal mesenchyme cells in the control group and RA10D cleft palate group on days E13.5, E14.5, and E15.5. ki67 immunofluorescence detection of cell proliferation levels in the palatal mesenchyme of embryonic mice in the control and RA10D/R zA12D cleft palate groups on days E13.5 (**B**), E14.5 (**C**), and E15.5 (**D**) (green: ki67, blue: DAPI) (10× magnification). (**E**) Immunohistochemical detection of METTL14 and PCNA expression in the palatal mesenchyme of embryonic mice in the control group and RA10D cleft palate group. n.s is considered not statistically significant, * means *p* < 0.05, ** means *p* < 0.01, *** means *p* < 0.001, **** means *p* < 0.0001.

As shown in Figure 4A, we found that the phospho-mTOR/mTOR ratio and PCNA expression in the RA10D group were significantly lower than those in the control group on days E13.5–E15.5 (*p* < 0.05), indicating that atRA inhibited the cell proliferation ability of the palatal mesenchyme. The RA10D group had lower CyclinD1 and higher P21 than the control group, with statistical differences (*p* < 0.05), indicating that atRA induced abnormal expression of key proteins in the G1/S phase of the mesenchyme cells of the palatal shelves in cleft palate mice. Proliferation marker ki67 immunofluorescence staining was used to evaluate the cell proliferation levels in the palatine process mesenchyme during E13.5–E15.5 in the control and cleft palate groups. The Ki67 positive level of the RA10D group was significantly lower than that of the control group at the E13.5–E15.5 stages (*p* < 0.0001) (Figure 4B,D). The ki67 level of the RA12D group was only inhibited on day E13.5 (*p* < 0.001), and there were no significant differences in the other periods compared with the control group. According to our previous findings, the RA12D group showed no significant changes in m^6^A levels or palatal mesenchymal cell proliferation levels compared with the control group during the critical period of palate development. The purpose of this study was to explore the effect of m^6^A modification on the proliferation of palatal mesenchymal cells, so we focused on the RA10D group in subsequent experiments. It can be concluded that in the RA10D cleft palate group, the m^6^A methylation modification level in the palatine process mesenchyme of cleft palate mice was increased and the cell proliferation was inhibited. At the same time, in order to understand the impact of atRA induction on the level of mesenchyme and epithelial apoptosis in the palatal tissue, and to observe whether METTL14 is related to regulating the MEE outcome, we performed immunofluorescence staining of cleaved-caspase 3. On days E13.5 and E14.5, cleaved-caspase 3 positive fluorescent signals were scattered in the palatal shelves in both groups (Supplementary Figure S2A,B). On day E15.5, the epithelium of the control group made contact and began to fuse, and apoptotic cells were enriched in the MES and epithelial triangle area. Apoptotic cells increased significantly in the RA10D mesenchyma, while the MEE was still double- or multi-layered without apoptosis (Supplementary Figure S2C). This experiment showed that apoptosis is one of the ways in which MES disappears. In the key stage of palate development (E13.5–E15.5), compared to the control group, the METTL14 of the palatal mesenchyme was increased in the RA10D cleft palate group (*p* < 0.01), ki67 was significantly reduced (*p* < 0.001), and there was no significant difference in caspase 3 expression (Supplementary Figure S2D). We used immunohistochemistry to detect the expression of METTL14 and proliferating nuclear antigen PCNA in the palatine process mesenchyme of the control group and RA10D group on days E13.5, E14.5, and E15.5 to verify the correlation between METTL14 and cell proliferation levels. As shown in Figure 4E, the expression of METTL14 was negatively correlated with the expression of PCNA (*p* < 0.05).

In summary, it can be seen that during the key stages of embryonic mice’s palate development (E13.5–E15.5), the level of m^6^A methylation modification and the expression of METTL14 in the palatine process mesenchyme of cleft palate were significantly higher than those of normal palatal shelves. This change may negatively regulate cell proliferation in the palatine process mesenchyme but has no significant correlation with the level of cell apoptosis. Therefore, we believe that METTL14 may affect cleft palate by mediating m^6^A methylation modification and regulating the cell proliferation level of the mesenchymal components of the palatal shelves.

### 2.5. Knockdown of METTL14 or Inhibition of m^6^A Methylation Modification Can Partially Rescue the Decline in Cell Proliferation Induced by atRA

**Figure 5:**
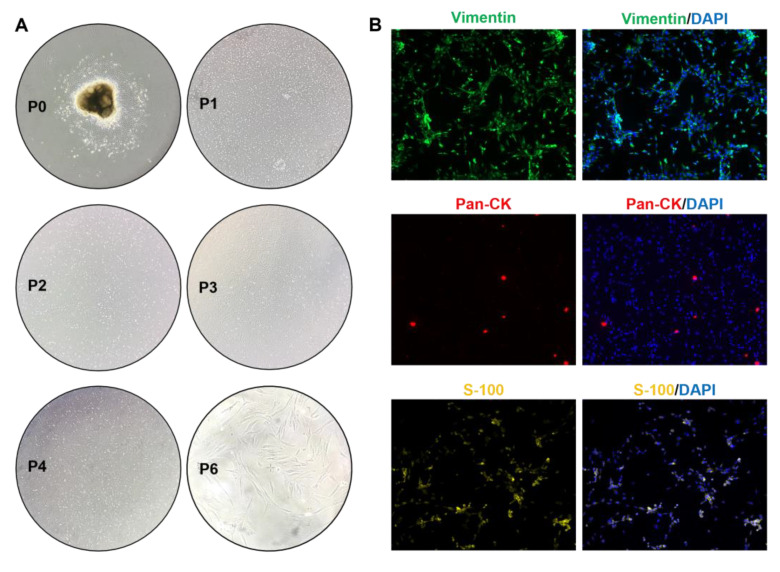

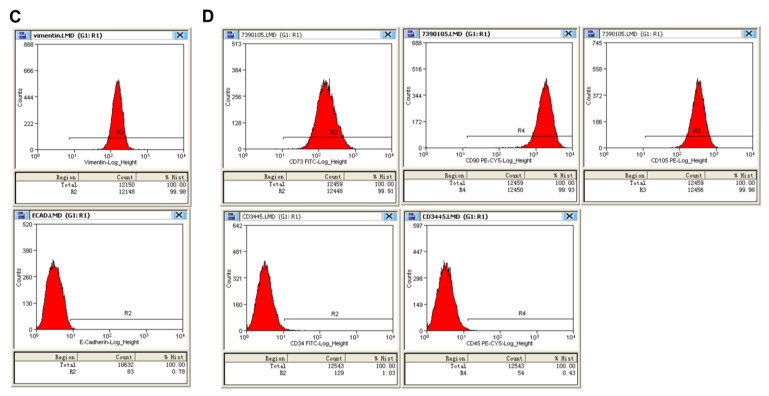
Morphological observation and identification of mouse embryonic palate mesenchymal cells (MEPMs). (**A**) Observation of MEPM cells under a light microscope. (**B**) Immunofluorescence identification of the source and characteristics of primary cells (green: vimentin, red: pan-CK, yellow: S-100, blue: DAPI) (10× magnification). (**C**) Flow cytometry to detect the positive signal amounts of the marker vimentin and epithelial marker E-cad in P0 generation MEPM cells. (**D**) Flow cytometry was used to detect the expression of cell stemness markers CD34, CD45, CD73, CD90, and CD105 in P2 generation MEPM cells.

Mouse embryonic palatal mesenchyme (MEPM) primary cells were isolated, and the morphology of the P0-P6 generation was observed under a stereomicroscope. Immunofluorescence and flow cytometry staining were used to identify the source, purity, and stemness of the cells. As shown in Figure 5A, the P0 MEPM cells grew adherently, and the P1-P4 generations proliferated rapidly and then gradually slowed down. ICC showed that the primary MEPM cells stained were vimentin (+), pan-CK (−), and S-100 (±) (Figure 5B). The positive rate of P1 cells detected by flow cytometry of the mesenchymal marker vimentin was 99.98%, and that of the epithelial marker E-cadherin was 0.78% (Figure 5C). According to the immunofluorescence identification results, most of the P1 cells were MEPM cells. In order to understand the characteristics of MEPM cells after passage, we stained the cells for stemness markers and detected them by flow cytometry. MSC markers (CD73, CD90, and CD105) were significantly positive in the P2 generation MEPM cells, while CD34 and CD45 were negative (Figure 5D). The above results indicate that MEPM cells have the characteristics of mesenchymal stem cells, with self-renewal, rapid proliferation, and multi-directional differentiation potential.

**Figure 6:**
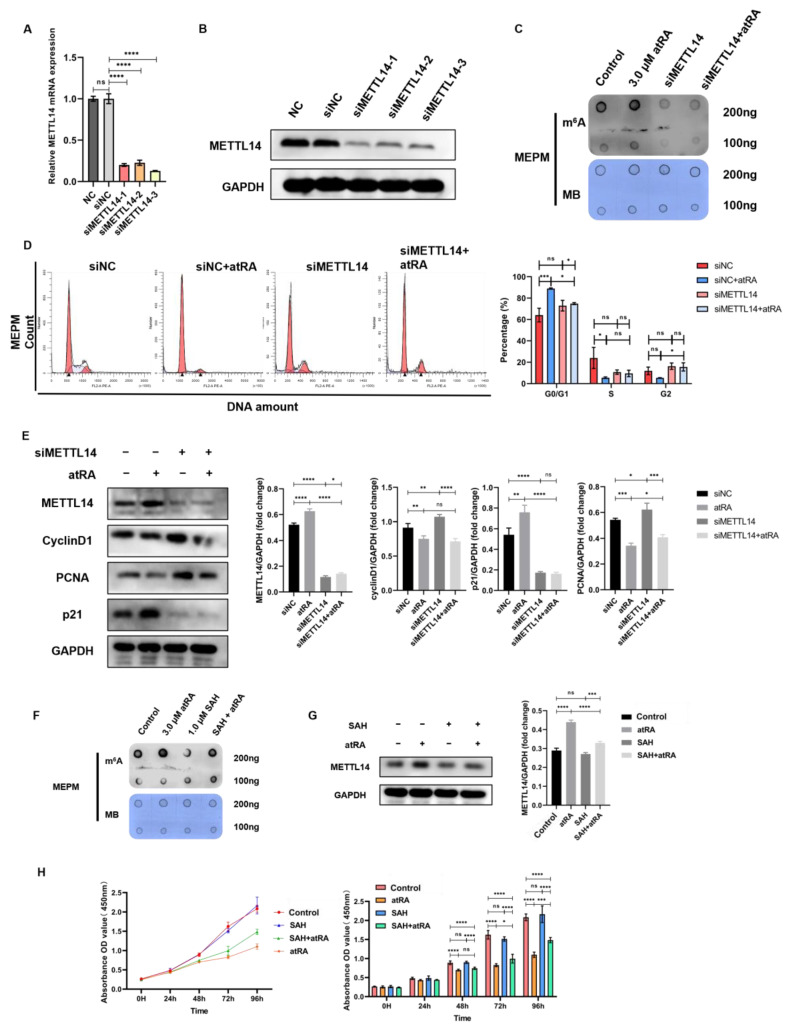
Knockdown of METTL14 or inhibition of m^6^A methylation modification can partially rescue the decline in cell proliferation induced by atRA. qRT-PCR (**A**) and Western blot (**B**) detection of the siRNA knockdown efficiency of METTL14. (**C**) Dot blotting detected the m^6^A methylation modification level of MEPM cells after treatment with siMETTL14 and atRA. (**D**) Flow cytometry to detect MEPM cell cycle changes after siMETTL14 and atRA treatments. (**E**) Western blot detected changes in MEPM cell cycle and proliferation-related proteins after siMETTL14 and atRA treatments. (**F**) Dot blotting detected m^6^A methylation modification level after SAH treatment of MEPM. (**G**) Western blot detection of METTL14 protein expression after SAH treatment of MEPM. (**H**) CCK8 experiment to detect cell proliferation after SAH and atRA treatments of MEPM. n.s is considered not statistically significant, * means *p* < 0.05, ** means *p* < 0.01, *** means *p* < 0.001, **** means *p* < 0.0001.

Based on our previous research, an in vitro MEPM culture system was constructed, and it was found that after induction with 3.0 μM atRA for 48 h, MEPM cells showed phenotypes similar to the palatine process mesenchymal cells of cleft palate mice (Supplementary Figure S3). Using siRNAs to knock down the expression of METTL14 in MEPM cells, qRT-PCR and Western blot were performed to detect the knockdown efficiency and determine the available sequences (Figure 6A,B). Dot blotting detects changes in m^6^A methylation modification levels in MEPM cells after siMETTL14 and atRA treatment alone and combined. It was found that interfering with METTL14 protein expression can affect the m^6^A methylation modification level of MEPM cells (Figure 6C). After combination with atRA treatment, since METTL14 cannot be expressed normally, the m^6^A methylation modification level did not change, confirming that the effect of atRA on m^6^A is dependent on the normal expression of METTL14. Compared to the atRA single-treatment group, the G0/G1 phase arrest of MEPM cells in the siMETTL14+atRA group was significantly alleviated (*p* < 0.05) (Figure 6D). This phenomenon showed that after interfering with the expression of METTL14, the effect of atRA on the cell cycle was smaller than the difference between the siNC group and siNC+atRA group, indicating that regulation of the cell cycle by atRA depends on the methyltransferase function of METTL14. Compared to the atRA group, the p-mTOR/total mTOR ratio, CyclinD1, and PCNA expression in the siMETTL14+atRA group were increased (*p* < 0.05), and p21 was significantly decreased (*p* < 0.05) (Figure 6E). It can be considered that interfering with METTL14 expression can affect the phosphorylation status of the downstream proliferation regulatory factor mTOR, nuclear proliferation antigen PCNA, G1/S phase key regulatory factor CyclinD1, and protein expression of PCNA, while atRA can regulate downstream target genes by affecting the m^6^A methylation modification levels and the expression of METTL14.

To understand whether METTL14 activity could affect atRA functions, we used the METTL3-METTL14 methyltransferase complex inhibitor SAH to simulate an environment in which m^6^A methyltransferase function is inhibited. We found that treatment of MEPM with 1.0 μM SAH could reduce the level of m^6^A methylation modification by inhibiting the formation of the METTL3-METTL14 methyltransferase complex without significantly affecting the expression level of METTL14 protein (Figure 6F,G). It can be seen from the CCK-8 experiment that there was no significant difference in the proliferation level of MEPM cells treated with SAH alone compared to the control group. At the same time, the 1.0 μM SAH + 3.0 μM atRA treatment can partially rescue the MEPM cell proliferation inhibition caused by atRA (*p* < 0.0001) (Figure 6H).

## 3. Discussion

Based on existing research, it is known that cleft palate can occur as a Mendelian syndrome as a monogenic disease, or as a non-syndromic disease due to chromosomal abnormalities or teratogen exposure. Developmental susceptibility and instability of genomic signals are important factors in the occurrence of malformations [22]. During the embryonic palate development period, a variety of cells are monitored through gene activation and inhibition pathways to regulate proliferation, differentiation, angiogenesis, accumulation of extracellular matrix, and apoptosis, and then form the appropriate sizes and morphology of the palate [23,24,25]. Changes in these factors may lead to insufficient tissue volume of the facial processes, thereby preventing contact or fusion. Accurate signaling pathways and regulatory mechanisms combined with a stable and harmless environment are essential for normal development. Researchers have found that small fluctuations in environmental factors may interfere with normal DNA synthesis and methylation modification of cells during the peak period of proliferation and differentiation through gene × environment interactions, leading to malformations [26,27]. Some studies have proven that environmental factors can affect the recruitment and competitive binding regulatory function of non-coding RNAs, thereby interfering with normal palate development [28,29]. This study aims to understand the role and possible regulatory mechanisms of RNA methylation in the occurrence of cleft palate in mice caused by environmental factors.

The use of chemical drugs as exogenous teratogens to induce cleft palate in mice is currently one of the most stable and effective methods for studying the molecular mechanism of environmental factors affecting palate development. These drugs include dexamethasone, retinoic acid, phenytoin, and tetrachlorodibenzo-p-dioxin (TCDD), etc. [30,31,32]. These drugs are widely used in clinical treatment. However, the teratogenic effects of such drugs can vary in phenotype due to differences in administration time, dosage, and solvent conditions [33], and this difference can be explained by epigenetic regulation [6]. Retinoic acid (RA) is a metabolite of vitamin A in organisms and can participate in regulating the directional differentiation of the reproductive system and early embryonic development in mammals [34,35]. It is currently recognized as the drug of choice for the treatment of acute promyelocytic leukemia (APL) and is also a common environmental teratogen [36,37,38]. This study uses atRA intragastric induction to establish a stable mouse cleft palate model, and is the first to explore the correlation between the imbalance of m^6^A methylation modification levels and the occurrence of cleft palate.

m^6^A is the most common and abundant form of RNA modification in eukaryotic cells. It mainly occurs on the conserved base sequence of [RRACH] (R = G/A, H = A/C/U) [39]. In the past two decades, scholars have begun to pay attention to the regulatory role of m^6^A methylation in embryonic stem cells and embryonic development [16,40,41,42]. The earliest report demonstrating that m^6^A is crucial for development was the observation that mice with knockout METTL3 were embryonically lethal. The METTL3-/- embryos appeared normal before implantation, but defects began to appear after implantation, and at embryonic day E8.5 the embryos were absorbed [43]. Later, some scholars knocked down METTL3 or METTL14, and the self-renewal ability of embryonic stem cells was significantly reduced [16]. They believed that m^6^A modification affected the mRNA stability of embryonic stem cell development regulators. Transcription factor Smad2/3 participates in TGFβ signaling to regulate normal development of the embryonic palatine process. Recently, some scholars have found that Smad2/3 can negatively regulate the proliferation of embryonic stem cells and affect their biological functions by recruiting and directly binding to the m^6^A methyltransferase complex to affect the expression of the downstream gene NANOG [44]. m^6^A is a reversible epigenetic modification that changes dynamically at every stage of embryonic development [45,46]. Researchers found that m^6^A sites were mainly enriched in embryonic development and cell proliferation pathways at E13, while m^6^A sites were mainly enriched in muscle differentiation- and growth-related pathways at E19 [47]. In this study, we paid more attention to the dynamic changes in m^6^A levels and METTL14 in palatal mesenchymal cells when cleft palate occurs. After our detection of m^6^A “writers” and “erasers” in the palatine process mesenchyme of cleft palate mice, it was found that the m^6^A methyltransferase METTL14 was abnormally elevated throughout the entire period of palate development, and was positively correlated with the m^6^A methylation modification level and negatively correlated with cell proliferation. In this study, we focused on the impact of m^6^A on the proliferation of palatal mesenchymal cells; the RA12D group showed no significant differences in m^6^A levels and cell proliferation levels compared with the blank control group during the critical period of palate development. Considering the aim of our study, we finally focused on the RA10D group. As we knew, METTL14 serves as an essential structure in the m^6^A methyltransferase complex and assists METTL3 in mediating reversible m^6^A methylation. Studies have confirmed that in zebrafish models, mettl3-knockdown embryos exhibited abnormal craniomaxillofacial malformation syndrome during embryonic development, and low expression of METTL3 inhibits the proliferation of BMSC, human embryonic palatal mesenchymal (HEPM) cells, and human dental pulp stromal cells (DPSCs) in vitro [17]. In our study, we used siRNA to interfere with METTL14 gene expression and SAH to inhibit the function of the METTL3-METTL14 methyltransferase complex and aimed to explore the effect of METTL14 on MEPM cell proliferation inhibition and G0/G1 phase arrest in an atRA-induced cleft palate mouse model. The results showed that the regulation of m^6^A by atRA depends on the correct and accurate expression and methyltransferase function of METTL14, and abnormal changes in METTL14 can directly affect the expression of downstream proliferation and cell cycle-related proteins.

Finding suitable activity inhibitors to antagonize the teratogenic effects of environmental factors is part of the research directions in the etiology and preventive treatment of cleft palate. It is universally acknowledged that folic acid (FA) can prevent fetal neural tube defects and cleft lip and palate in the pregnancy preparation and early pregnancy periods [48,49]. Abnormal DNA methylation induced by environmental factors such as PM2.5 can lead to cardiac malformations in zebrafish embryos, and the application of folic acid can play an antagonistic and protective role [50]. Based on the results of previous studies, finding a m^6^A methylation modification inhibitor and exploring its antagonistic effects on atRA has been identified in this article as one of the issues to be studied. S-adenosyl-L-homocysteine (SAH) is derived from S-adenosyl-L-methionine (SAM) through demethylation and can be used as an inhibitor of the METTL3-METTL14 methyltransferase complex [51]. SAH, with an IC50 value of 0.9 ± 0.1 μM, has no significant effect on the activity of another m^6^A methyltransferase, ALKBH5 [52]. Studies have found that application of SAH can promote the apoptosis and autophagy of inflammatory chondrocytes in vitro, and can aggravate degeneration of the chondrocytes and subchondral bone in a temporomandibular joint osteoarthritis (TMJOA) mouse model in vivo [53]. SAH can effectively inhibit the function of the m^6^A methyltransferase complex, thereby blocking the m^6^A methylation modification process and inhibiting the m^6^A methylation modification level.

m^6^A modifications mostly occur on the conserved base sequence of [RRACH] (R = G/A, H = A/C/U), and the enzymes can perform their function by recognizing the relevant sites and binding to them. Therefore, according to the cDNA or mRNA sequence of the gene, the potential modification site of the target gene can be determined by searching for “GGACU”, “GGACA”, “GGACC”, “AGACU”, and other combinations that conform to the [RRACH] motif rules [54]. Single-nucleotide polymorphisms (SNPs) can also cause changes in m^6^A modification [55,56]. By using genome-wide association studies (GWASs), many studies have screened out SNP sites that are critical to the risk of cleft lip and palate [57,58,59]. Searching the disease keywords “cleft palate” in the m^6^A Var database [60] showed that many recognized cleft palate susceptibility gene sequences have SNP changes that are converted into m^6^A modification sites, such as rs187379424 (G > A) of IRF6, rs886055457 of SUMO1 (T > C), and rs1058213 (G > A) of TGFα, etc., indicating that the m^6^A methylation modification level may change accordingly after SNPs occur in such genes. These scientific inferences are still not verified by further experiments. Therefore, in future studies, we will pay more attention to finding m^6^A modification sites or potential m^6^A-SNPs in susceptible genes to better understand the mechanism of m^6^A methylation modification in cleft palate.

## 4. Materials and Methods

**Mice**

A total of 66 C57BL/6J mice were purchased from Sun Yat-sen University and raised in an SPF environment in the standard experimental animal center. Females (10–12 weeks, 20–25 g) were mated with mature males (24–30 g) in the morning, and the time when a vaginal plug was detected was recorded as E0 (embryonic day 0). Female mice that were confirmed to be pregnant were randomly assigned to the experimental and control groups. Female mice with a distended abdomen and whose weight had increased by 5 g in 10 days from the day of mating were considered pregnant. This experimental design strictly followed the 3R principle (reduction, replacement, refinement). Pregnant females at E10/E12 were administered intragastrically 100 mg/kg of atRA (Sigma-Aldrich, St. Louis, MO, USA) dissolved in corn oil, whereas the control animals were administered the same volume of corn oil at the same time. Pregnant mice were sacrificed by cervical dislocation after anesthesia on E13.5, E14.5, E15.5, and E18.5. The fetuses were used for subsequent experiments. All of the animal experiments complied with the Animal Research: Reporting of in vivo Experiments (ARRIVE 2.0) guidelines [61] and were carried out in accordance with the National Research Council’s Guide for the Care and Use of Laboratory Animals. This animal experiment protocol was reviewed and approved by the Animal Ethics Committee of Sun Yat-sen University and complies with the relevant regulations on experimental animal welfare and ethics of the US NIH (approval number: SYSU-IACUC-2021-000433).

**Collection of palatal shelves and isolation and culture of primary MEPM cells**

Pregnant mice were sacrificed by cervical dislocation at E13.5/14.5/15.5/E18.5 days. Embryonic mouse heads and palatal shelves were separated using microscopic instruments under sterile conditions (Supplementary Figure S4). The heads were fixed with 4% paraformaldehyde fixative for the staining experiments. The palatal shelf was temporarily stored in 1% dual antibiotic PBS at 4 °C. E13.5 palatal shelves were incubated in 1 U/mL of dispase II (4942078001, Roche, Basel, Switzerland) serum-free DMEM/F12 culture medium at 37 °C for 1 h, and then the epithelium was peeled off. The remaining palate mesenchymal tissue was cut into 1 mm^3^ pieces and sieved through a 100 μm sterile cell sieve (MilliPore, MERCK, Billerica, MA, USA) and then dissociated into a cell suspension for MEPM primary cell culturing in subsequent experiments.

**Histological staining**

Fresh embryonic mouse heads were soaked in 4% paraformaldehyde for 24 h and dehydrated in a gradient of ethanol and fixed in paraffin wax. Then, 4 μm-thick paraffin sections were prepared for HE (Jiancheng BI, Nanjing, China) and immunohistochemical staining. The sample processing and staining steps were performed according to our previous protocol [62]. The primary antibodies were anti-METTL14 (NBP1-81392 NOVUS, Chesterfield, CO, USA), anti-PCNA (71395SF CST, Danvers, MA, USA), anti-ki67 (ab16667, abcam, Cambridge, UK), and anti-cleaved-caspase 3 (9579S CST, Danvers, MA, USA). IHC used DAB (DAB-0031, Maixin Biotechnology, Fuzhou, China) and HRP-goat anti-rabbit IgG secondary antibody (G1213 Servicebio, Wuhan, China). An Axio Imager Z2 optical microscope (Carl Zeiss, Oberkochen, Germany) was used for observation and an Aperio AT2 scanner (Leica, Wetzlar, Germany) was used to record the whole slide. For immunofluorescence, DAPI (C0065-10, Solarbio, Beijing, China), Alexa Fluor^®^ 488 Anti-rabbit IgG (H + L) (4412S CST, Danvers, MA, USA) and an anti-fluorescence quenching seal tablet (P36983 Thermo Fisher, Waltham, MA, USA) were used. An Axio Vert.A1 inverted fluorescence microscope (Olympus) and a LSM780 laser confocal microscope (ZEISS) were used to observe and photograph.

**RNA extraction and Quantitative reverse transcription–PCR (qRT-PCR)**

Total RNA was extracted from the palatal shelves and MEPM cells using RNAzol^®^RT (RN190 Molecular Research Center, Cincinnati, OH, USA). Then, reverse transcription was performed using a PrimeScript RT reagent kit (RR036A Takara, Shiga, Japan). Finally, SYBR Green Master Mix (11201ES08 Yeason, Shanghai, China) was added and real-time PCR was performed using a Roche LightCycler96 system (Roche Diagnostics, Burgess Hill, UK). Relative gene expression levels were calculated using the 2^−ΔΔCt^ method, with the level normalized to GAPDH. The design and selection of primer sequences were based on previous literature, and the specific primer sequences against targeted genes are listed in Supplementary Table S1 and were synthesized and provided by Shanghai Jierui Bioengineering Co., Ltd. (Shanghai, China).

**RNA-seq**

Tissue and cell RNA extraction and sequencing were performed by Shanghai Meiji Biomedical Technology Co., Ltd. (Shanghai, China). GO analysis and KEGG pathway enrichment analysis were performed on the obtained data to screen for significantly differentially expressed genes and potential pathways.

**Protein extraction and Western blot**

RIPA lysis buffer (CW2333S CWBIO, Beijing, China) containing a protease inhibitor and a phosphatase inhibitor was used to extract total protein from palatal shelves and MEPM cells. A BCA Protein Assay Kit (CW0014S CWBIO, Beijing, China) was used for quantification and standardization. Total protein (25 μg/20 μg) was separated by 10% SDS-PAGE and transferred to a PVDF membrane (0.22 μm Millipore, MERCK, Billerica, MA, USA). After blocking with 5% BSA (V900933 Sigma-Aldrich, MERCK, Billerica, MA, USA), the membrane was incubated with primary antibodies at 4 °C overnight and HRP-conjugated secondary antibody at room temperature for an hour. The antibodies are listed in Supplementary Table S2. Bands were visualized using an ECL kit (WBKLS0500 Millipore, MERCK, Billerica, MA, USA) and observed with a Bio-rad ChemiDoc XRS+ (Thermo Fisher Scientific, Waltham, MA, USA).

**Dot blot**

RNA samples from the embryonic palate tissues or MEPM cells were used to detect the binding ability of m^6^A antibodies in different models. An equal volume of SSC buffer (S196387 Aladdin, Shanghai, China) was used to balance all RNA samples to a concentration of 50 ng/μL. Then, 4 μL/2 μL RNA was transferred to a positively charged nylon Hybond-N+ membrane (RPN303B Solarbio, Beijing, China) and cross-linked with UV light (125 mJoule/cm^2^ at 254 nM), incubated with a recombinant m^6^A antibody (ab284130 abcam, Cambridge, UK), and stained with methylene blue (M9140 Sigma-Aldrich, MERCK, Billerica, MA, USA). ECL (WBKLS0500 MilliPore, MERCK, Billerica, MA, USA) was used to expose and Bio-rad ChemiDoc XRS+ (Thermo Fisher Scientific) to observe the imaging.

**Immunofluorescence identification of primary MEPM cells**

The P0 generation MEPM cells were digested and resuspended in a special confocal culture dish (diameter of 20 mm) with 1 × 10^6^ cells/1 mL. After the cells had adhered, they were fixed with 4% paraformaldehyde and incubated with vimentin, pan-CK, and S-100 monoclonal antibody (Supplementary Table S2), and then the currently configured FITC fluorescent-labeled secondary antibody and DAPI were used for staining. An LSM780 laser confocal microscope was used to photograph and identify the source of MEPM based on the positive rate.

**Flow cytometry for cell purity, stemness, and cell cycle detection**

MEPM cells were digested with TrypLE™ Express (Gibco, NY, USA) and collected. The cell surface was stained with 100 μL staining buffer and 1 μL labeled flow cytometry antibodies (Supplementary Table S3). The cell cycle detection was performed by using a Cell Cycle Staining Kit (CCS012 MultiSciences, Hangzhou, China). All the samples were incubated in the dark according to the protocol, and then a Cytoflex flow cytometer (Beckman Coulter, Brea, CA, USA) or BD LSR Fortessa flow cytometer (Beckman Coulter, Brea, CA, USA) was used for detection and analysis. ModfitLT 5 saainc software (VeritySoftwareHouse Inc., Topsham, ME, USA) was used to fit and draw the cell cycle, cell purity, and stemness curves.

**Preparation of special culture medium**

Absolute ethanol was used as the solvent to dissolve all-trans retinoic acid (atRA, R2625 Sigma-Aldrich, MERCK, Billerica, MA, USA), and DMSO (D2650 Sigma-Aldrich, MERCK, Billerica, MA, USA) as the solvent to dissolve S-adenosyl-L-homocysteine (SAH, S7868 Selleck, Houston, TX, USA). Then, the concentration to 10 mM was adjusted as the mother solution. DMSO and ethanol were used as solvents and blank control intervention factors in the prepared culture medium so that the concentration did not exceed 0.1%. A specific drug culture medium with DMEM/F12 culture medium containing 10% FBS or that was serum-free was calculated and prepared according to the concentration gradient, then mixed well and used immediately.

**Cell Proliferation Assay**

Cell proliferation was detected by using a Cell Counting Kit-8 (40203ES80 Yeason, Shanghai, China). MEPM cells in the logarithmic growth phase (2500 cells/well) were seeded in a 96-well plate and cultured by special medium. A Multiskan Spectrum microplate reader (BioTek, Lexington, MA, USA) was used to detect the OD value of the cells at each time point (0 h, 12 h, 24 h, 48 h, and 72 h) at an absorbance wavelength of 450 nm.

**RNA interference**

METTL14 siRNA was synthesized by Guangzhou Ribobio Biomedical Technology Co., Ltd. (Guangzhou, China). The gene sequences were as follows:

siMETTL14-1: 5′-TTGAAGAATACCCTAAACT-3′

siMETTL14-2: 5′- AGATGAACAGAGGGAGATT-3′

siMETTL14-3: 5′- GCATTGGTGCTGTGTTAAA-3′

MEPM cells in the logarithmic growth phase were evenly seeded in a 6-well plate, using Opti-MEMTM (31985-062 Gibco, Grand Island, NY, USA) and LIPOFECTAMINE RNAimax (13778150 Gibco, Grand Island, NY, USA) as the transfection system, and mixed with siRNA in the plate and incubated. Cellular RNA and protein were collected after 24 h and 48 h, respectively, for subsequent experiments.

**Statistical analyses**

GraphPad Prism 8.0 software (www.graphpad.com, accessed on 10 June 2020) was used to draw histograms and conduct statistical analysis. The Shapiro–Wilk test was used to test normal distribution. A two-tailed unpaired or paired Student’s *t*-test was used to analyze statistical differences between two groups. Data statistics for three groups were analyzed using one-way ANOVA with Tukey’s multiple comparison tests. The correlation between genes was analyzed using Pearson’s correlation. All reported *p* values are two-tailed, and *p* < 0.05 is considered statistically significant. n.s is considered not statistically significant, * means *p* < 0.05, ** means *p* < 0.01, *** means *p* < 0.001, and **** means *p* < 0.0001.

## 5. Conclusions

In the cleft palate mouse model, the level of m^6^A methylation modification in the palatine process mesenchyme increases and the level of cell proliferation decreases. These changes are caused by the abnormally elevated m^6^A methyltransferase METTL14 that regulates the cell cycle and proliferation.

## Figures and Tables

**Table 1:** Comparison of the incidence of cleft palate in embryonic mice between the cleft palate groups and the control group.

Group	Total Number of Embryonic Mice	Number of Stillbirths/ Absorptions	Number of Embryonic Mice with Cleft Palate	Survival Rate (%)	Incidence of Cleft Palate (%)
RA10D ^&^	136	25	108	81.6%	97.3%
RA12D	116	8	92	93.1%	85.2%
Control *	105	0	0	100.0%	0

^&^ RA10D and RA12D are the experimental groups of embryonic mice induced with retinoic acid on the 10th (E10) or 12th (E12) embryonic day, respectively. * According to the “Reduction” principle in the 3R principles of the animal experiment design, we reduced the number of experimental animals in the blank control group while ensuring the reliability of the experiment.

## Data Availability

The experimental data that support the findings of this study will be shared on reasonable request to the corresponding authors.

## Supplementary Materials

The following supporting information can be downloaded at https://www.mdpi.com/article/10.3390/ijms25084538/s1.

## Abbreviations

CL/P: cleft lip with or without palate; CPO, cleft palate only; atRA, all-trans retinoic acid; MEPM, mouse embryonic palate mesenchymal; METTL14, methyltransferase 14; MEE, medial edge epithelial; MES, medial epithelial seam; PS, palatal shelf; SAH, S-adenosyl-L-homocysteine; SNP, single-nucleotide polymorphism.
