# Supplementary material for: Retinoic Acid Upregulates METTL14 Expression and the m6A Modification Level to Inhibit the Proliferation of Embryonic Palate Mesenchymal Cells in Cleft Palate Mice
Source: Int J Mol Sci. 2024 Apr 20;25(8):4538. doi: 10.3390/ijms25084538 (PMC11050043; doi:10.3390/ijms25084538)
Supplement: Supplementary file 1 [file ijms-25-04538-s001.zip › ijms-2948446-supplementary.pdf]

Supplementary Table S1

Table S1 The specific primer sequences against the targeted genes

Gene	Gene ID	Sequences
METTL3	56335	Forward：5′-AGCAGAGCAAGAGACGAATTATC-3′
		Reverse：5′-GGTGGAAAGAGTCGATCAGCA-3′
METTL14	210529	Forward：5′-CTGAGAGTGCGGATAGCATTG-3′
		Reverse：5′-GAGCAGATGTATCATAGGAAGCC-3′
FTO	26383	Forward：5′-TTCATGCTGGATGACCTCAATG-3′
		Reverse：5′-GCCAACTGACAGCGTTCTAAG-3′
ALKBH5	268420	Forward：5′-CGCGGTCATCAACGACTACC-3′
		Reverse：5′-ATGGGCTTGAACTGGAACTTG-3′
WTAP	60532	Forward：5′-GAACCTCTTCCTAAAAAGGTCCG-3′
		Reverse：5′-TTAACTCATCCCGTGCCATAAC-3′
GAPDH	14433	Forward：5′-AGGTCGGTGTGAACGGATTTG-3′
		Reverse：5′-TGTAGACCATGTAGTTGAGGTCA-3′

Supplementary Table S2

Table S2 List of the antibodies used for Western blot and immunofluorescence

Antibody name	Item number	Manufacturer
ki67	ab16667	abcam，UK
METTL14	NBP1-81392	NOVUS，USA
Cleaved Caspase-3	9579S	CST，USA
phospho-mTOR	5536S	CST，USA
mTOR	2983S	CST，USA
CyclinD1	2978S	CST，USA
PCNA	71395SF	CST，USA
p21 Waf1/Cip1	2947S	CST，USA
GAPDH	5171S	CST，USA
HRP- Goat anti-rabbit IgG secondary antibody	G1213	Servicebio, China
Anti-rabbit IgG (H+L), F(ab')2 Fragment (Alexa Fluor® 488 Conjugate)	4412S	CST，USA
Vimentin	5741S	CST，USA
pan-CK	ab264485	abcam，UK
S-100 A1	AF0251	Affinity, China

Supplementary Table S3

Table S3 List of the antibodies used for Flow Cytometry

Antibody name	Item number	Manufacturer
Vimentin	5741S	CST，USA
E-cadherin	3195S	CST，USA
PE/Cyanine7 anti-mouse CD34	119325	Biolegend，USA
APC/Cyanine7 anti-mouse CD45	103116	Biolegend，USA
APC anti-mouse CD73	127209	Biolegend，USA
Alexa Fluor® 700 anti-mouse CD90.2	105320	Biolegend，USA
APC anti-mouse CD105	120413	Biolegend，USA

Supplementary Figure S1

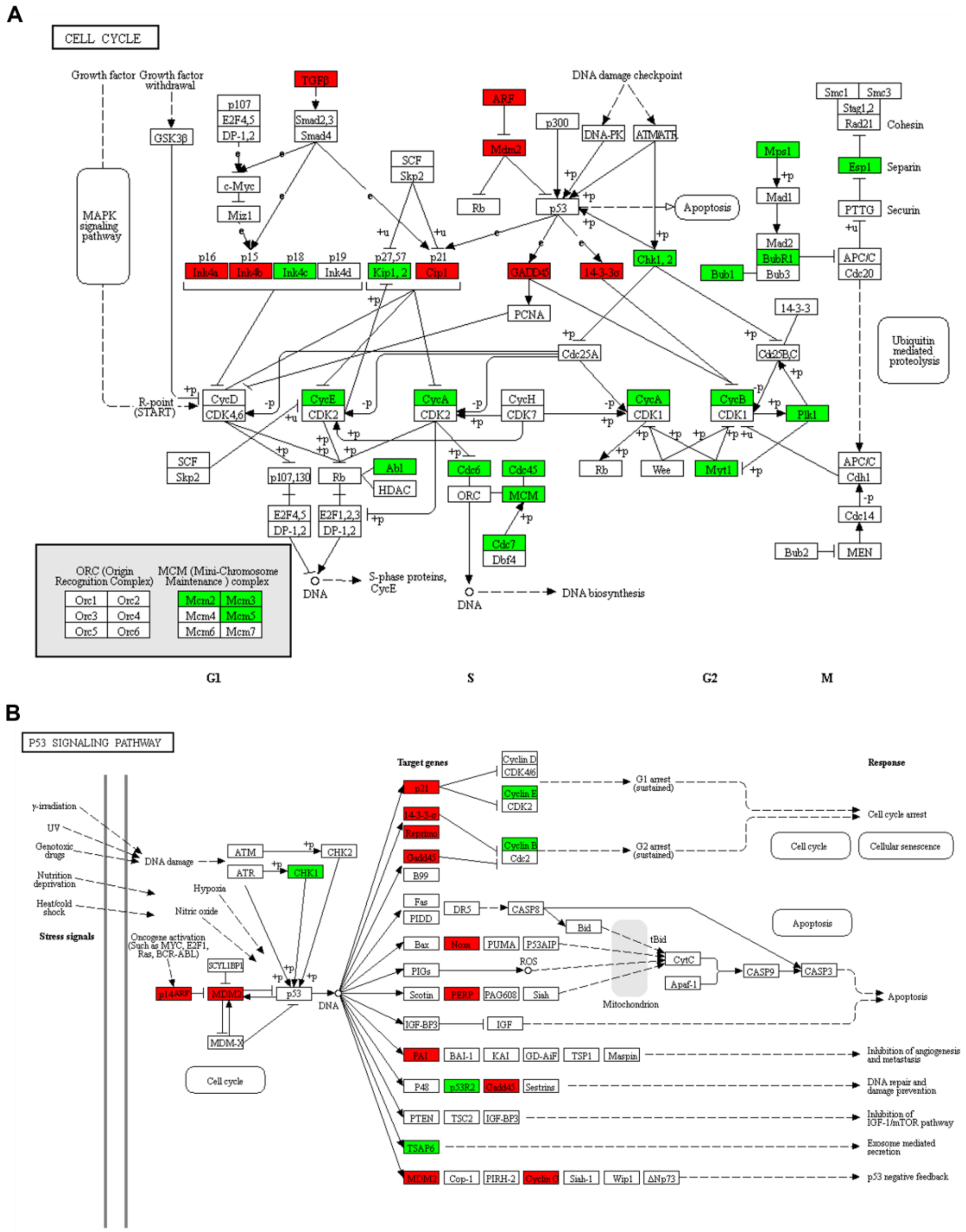

Figure S1. Schematic diagram of differentially expressed genes and enrichment pathway signal transduction in the palatal mesenchyme of embryonic mice on day E13.5 between the control group and the RA10D group. (A) Cell cycle-related signal pathway. (B)P53 signaling pathway.

Supplementary Figure S2

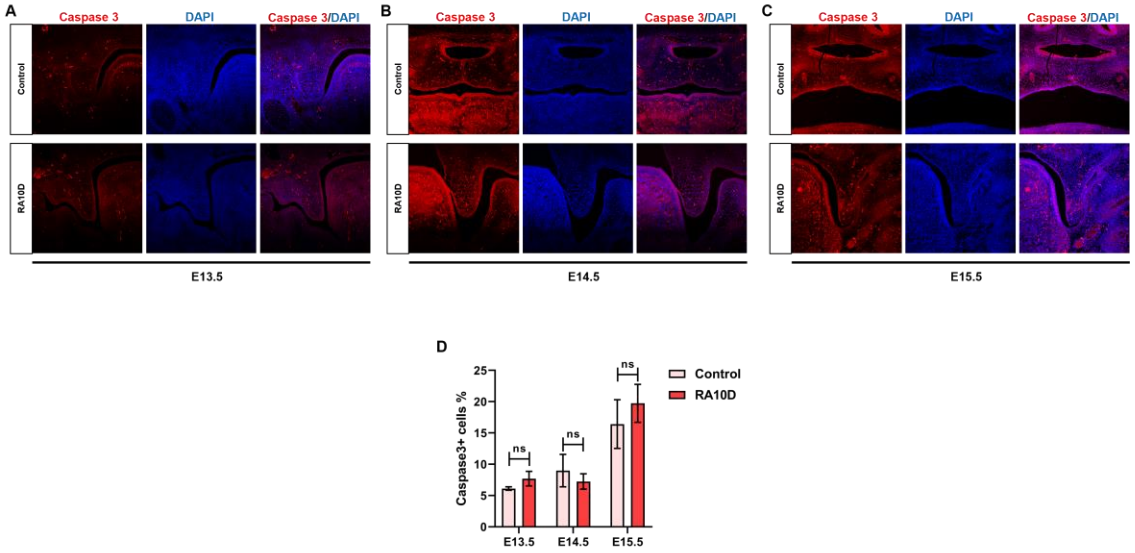

Figure S2. Immunofluorescence detection of apoptosis levels (cleaved-caspase 3 positive fluorescent signal rate) of palatal process mesenchyme and epithelial cells in the control group and RA10D cleft palate group in the key periods of palate development (10x magnification). (A)E13.5 day. (B)E14.5 day. (C)E15.5 day. (D)Semi-quantitative analysis of Caspase 3 immunofluorescence expression in the control group and RA10D group. n.s is considered not statistically significant, * means P<0.05, ** means P<0.01, *** means P<0.001, **** means P<0.0001.

Supplementary Figure S3

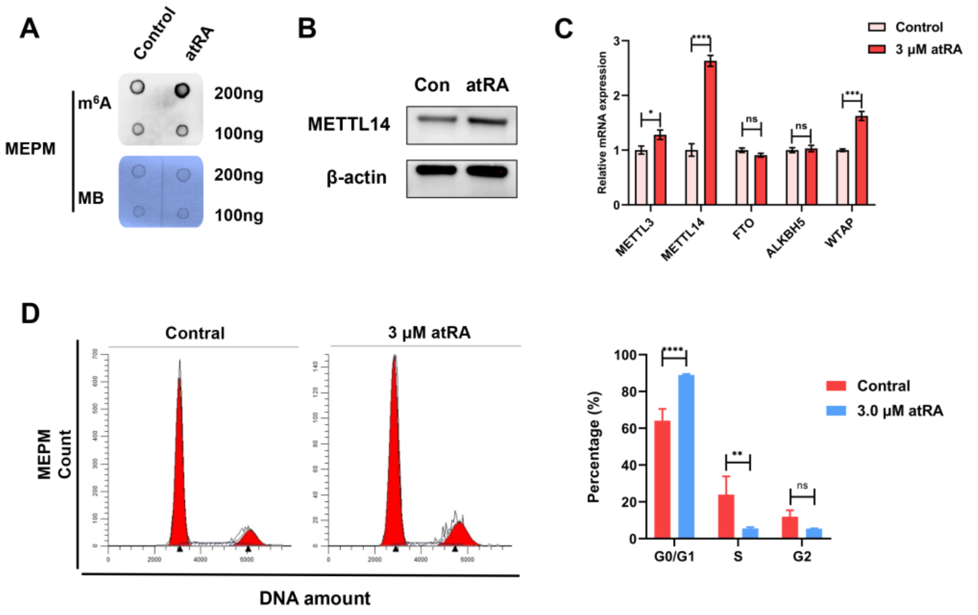

Figure S3. MEPM cells cultured by 3.0 μM atRA for 48 hours showed the same phenotype and gene expression level as the palatal process mesenchyme cells of embryonic cleft palate mice. (A) Dot blotting to detect m6A methylation antibody binding level. (B) Western blot detection of METTL14 expression. (C) qRT-PCR detection of mRNA expression of m6A 'Writers' and 'Erasers'. (D) Flow cytometry to detect cell cycle. (n.s is considered not statistically significant, * means P<0.05, ** means P<0.01, *** means P<0.001, **** means P<0.0001.)

Supplementary Figure S4

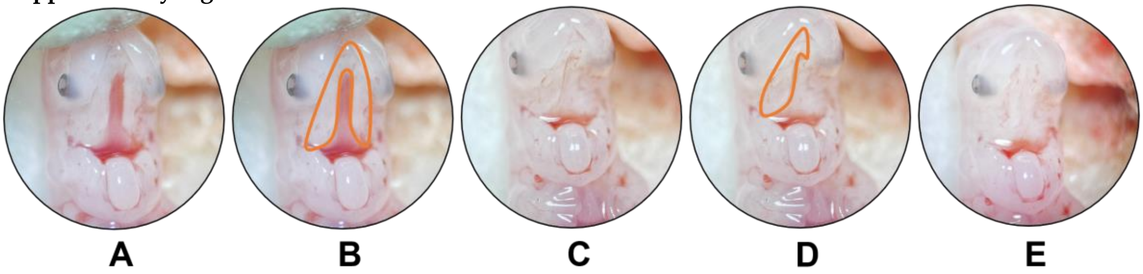

Figure S4. Obtaining palatal process tissue from E13.5day embryonic mice through micromanipulation. (A-B) Cutting along the angulus oris to separate the upper and lower jaws and expose the bilateral palatal processes. (C-D) Microsurgery to separate and remove the palatal processes. (E) View of the remaining tissue after the bilateral palatal processes are completely obtained.
